# Obituary: Dr. Mark Andrew Batzer: October 26, 1961 - January 21, 2026

**DOI:** 10.1186/s13100-026-00408-w

**Published:** 2026-08-07

**Authors:** Prescott L. Deininger, Stephen T. Sherry, Jerilyn A. Walker, Jinchuan Xing, David A. Ray, Richard Cordaux, Shurjo K. Sen

**Affiliations:** 1 Tulane Cancer Center, Tulane University, New Orleans, LA 70112 USA; 2 National Library of Medicine, National Institutes of Health, Bethesda, MD 20892 USA; 3 Department of Biological Sciences, Louisiana State University, Baton Rouge, LA 70803 USA; 4 Department of Genetics, Rutgers, The State University of New Jersey, Piscataway, NJ 08854 USA; 5 Department of Biological Sciences, Texas Tech University, Lubbock, TX 79424 USA; 6 Université Paris-Saclay, CNRS, IRD, UMR Évolution Génomes Comportement Écologie, Gif-sur-Yvette, 91190 France; 7 National Human Genome Research Institute, National Institutes of Health, Bethesda, MD 20982 USA

**Figure:**
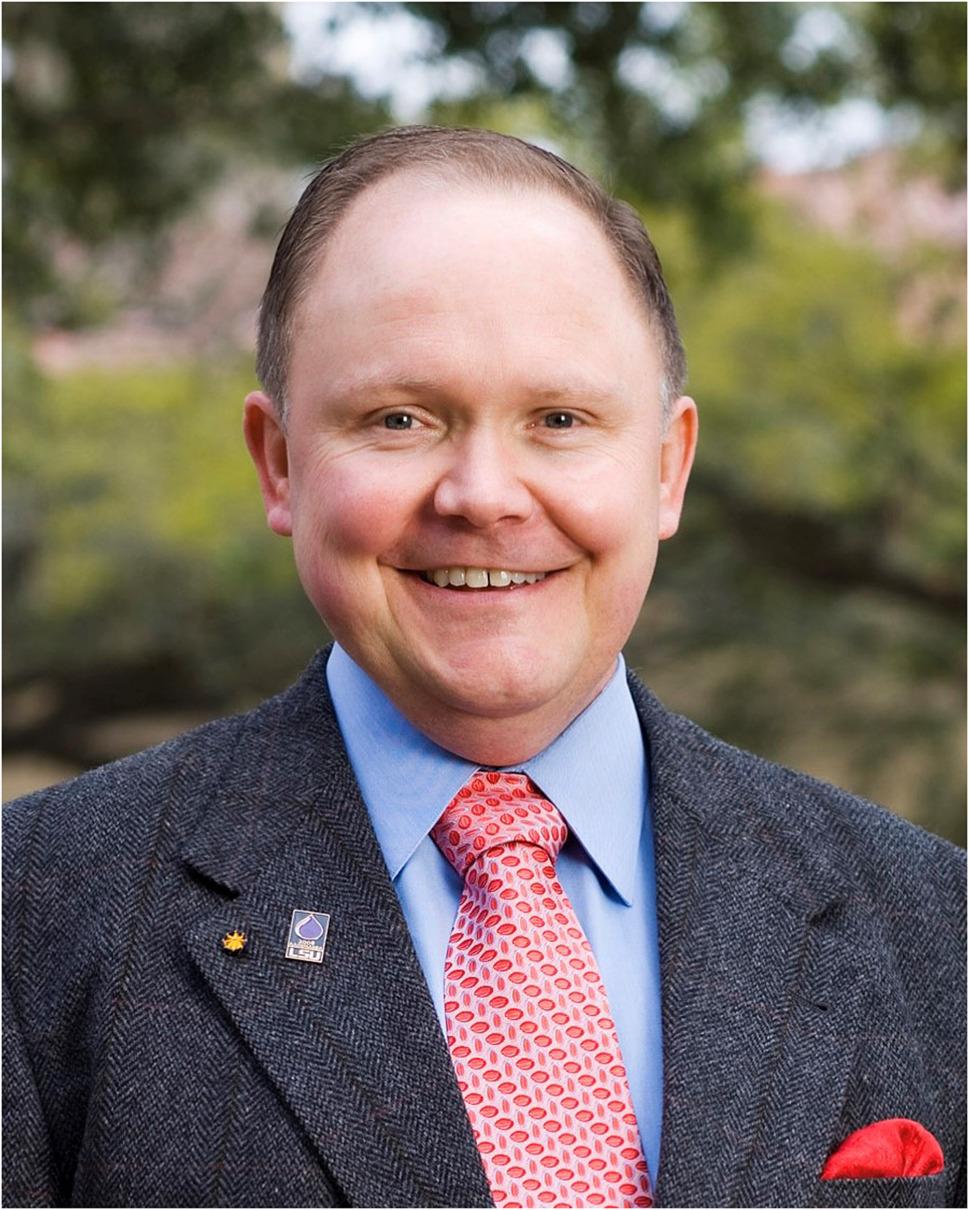

In January 2026, the many friends, collaborators, and trainees of Dr. Mark Batzer were left with a hole in their hearts at his untimely passing. Mark was a giant in the world of mobile DNA and a truly irreplaceable character. No one whose life intersected with his was left untouched. Through this story, we share Mark’s scientific and personal legacy with the community he loved most—his fellow mobile DNA element scientists.

Mark was the only child of Douglas and Marie Batzer. Born prematurely, he entered the world with the same resilience that defined the future course of his remarkable career. Encouraged by an inspiring teacher, he discovered a passion for biology early on and once considered becoming a veterinarian, but after the painful loss of his beloved dog, he turned his focus to genetics. Hardworking since his childhood, Mark showed his trademark determination since the beginning of his career, which started as a stock clerk at Winn-Dixie. Although he attended a high school where few students pursued college, he pursued and gained an undergraduate spot at Michigan State University, followed by a Ph.D. from Louisiana State University (LSU), in Baton Rouge under the mentorship of Bill Lee, working in the field of Drosophila genetics. He got his first introduction to mobile DNA as a postdoctoral fellow working with Prescott Deininger at LSU Health Sciences Center (LSUHSC) in New Orleans. In a lucky twist of fate for mobile DNA science, Mark nearly turned down the postdoctoral offer from Prescott for another one to work on the relatively young but high-profile Human Genome Project. Thankfully, he changed his mind quickly, to stay near the love of his life and his future wife, Pamela Richard (Pam, to all who know her), as she attended medical school at LSU. Throughout his later years, Mark stated that he never regretted that decision, as it led to him gaining the two things he cherished the most—Pam and a lifelong attachment to mobile DNA.

During his postdoctoral fellowship, Mark helped uncover the basic subfamily structure for *Alu* elements, shortly after earlier research in the Deininger lab had identified the first human-specific insertions. Mark created methods for the identification of more such subfamilies and elucidated the polymorphic nature of *Alu* insertions in a population sense, ultimately leading to the development of the “master gene” model for *Alu* amplification [1]. During this period, it also quickly became obvious that Mark was going to be a future leader for this field, as apart from his own prodigious research output, he thrived on the opportunity to help guide and mentor younger members of the Deininger laboratory. His training with Prescott eventually evolved into a lifelong hybrid personal friendship/scientific collaboration, eventually leading them to publish over 50 papers and travel to countless locations together.

Following his postdoctoral training, Mark moved to the Lawrence Livermore Genome Center where he became a Research Scientist and did fundamental work developing cutting-edge techniques for the still-nascent science of genomics. This, combined with his prior postdoctoral expertise in using high-throughput approaches for studying *Alu* polymorphisms, prepared him well for his subsequent distinguished career using these elements to explore questions in comparative genomics and population biology. Notably, during this time, along with Prescott and Carl Schmid, Mark co-organized the first scientific meeting focused primarily on mobile elements (held at Lake Tahoe in 1994) which is often considered the precursor to the very successful FASEB mobile element conference series that continues to this day. Arising from this meeting was an agreement on a standardized nomenclature for *Alu* elements [2]. Mark used his unparalleled social skills to lead and moderate the contentious discussion at the meeting on the harmonized naming of *Alu* elements, a difficult task far beyond what this brief description can do justice to. This publication was instrumental for "corralling in" the growing magnitude of future SINE mobilization discoveries. Beyond human mobile elements, the standardized nomenclature continues to be used today across primate genomes (e.g. bonobo, orangutan, rhesus, gibbon and tamarin). Most recently, it was applied to newly discovered Platy-1 SINE subfamilies in the howler monkey genome (published earlier this year in *Mobile DNA* as the final article from the Batzer laboratory) [3].

In 1995 Mark was recruited back to LSUHSC in New Orleans as an assistant professor, bringing him and Pam back to Louisiana. With his typical urgency and drive, he rapidly established his laboratory as a leading center for the empirical study of human *Alu* polymorphisms, building datasets of insertion polymorphisms across geographically diverse populations that attracted a succession of collaborators. Stephen Sherry, a postdoctoral fellow from 1996 to 1998, applied coalescent theory to Mark's *Alu*Ya5 datasets to produce one of the first estimates of human effective population size from mobile element data. When he left for NLM/NCBI, Mark’s sustained partnerships with population geneticists Henry Harpending and Lynn Jorde continued to generate productive work, with Mark and Lynn alone publishing some 25 papers together through 2013. Throughout this period Mark trained a long series of Ph.D. students and postdocs (beginning with Margaret Deangelis, his first doctoral trainee, who did foundational gene mapping work for Usher Syndrome in his lab) and cementing the foundation of his lifelong reputation as both a rigorous empiricist and generous mentor.

While at LSUHSC, around 2000, Mark received an offer from the LSU’s Department of Biological Sciences, for plentiful lab space in their newly constructed Life Sciences Annex building (LSA) if he would consider moving the Batzer Lab from New Orleans to the Baton Rouge campus. Given the growing size of his group and the possibilities for expanding his lab’s experimental capabilities, the offer was irresistible and he accepted. Unfortunately, construction of the LSA was not quite finished when he arrived in Baton Rouge, and for a brief window the Batzer Lab was essentially homeless. Mark remained characteristically positive and soon the brand-new building had his team as its first new inhabitants – ready or not. It quickly became a home away from home for many brilliant young minds from around the globe, with lab members coming from 15 countries over the ensuing 25 years. The lab steadily grew to a peak size of over 25 members, and productivity accelerated rapidly as Mark mentored 20 doctoral students, 13 postdoctoral fellows, a master’s student and 70 undergraduate research scientists (who he would affectionately refer to as “the zygotes”, of whom at least 50 went on to medical school). Some of those who Mark called “kids” are now themselves in their 50’s, and together with > 100 members spanning the globe, they form the lasting Batzer Lab family.

At times Mark is referred to as “Mr. *Alu* element”, given his foundational role in understanding species-specific *Alu* retrotransposons across a broad variety of primate genomes. Building on his postdoctoral work that laid the framework for this field, Mark’s subsequent research drastically broadened the field of *Alu* element biology. Mark harnessed the great potential of using *Alu* insertions as informative markers and extended the research from basic genomic biology of retrotransposons to fundamental advances in their applications to population genetics, phylogenetics, and forensic science. Since his seminal postdoctoral work reporting the analysis of recently integrated, human-specific *Alu* elements which turned out to be nearly identical in sequence, Mark and Prescott proposed the “master gene” model of *Alu* evolution, which posits that amplification of a given *Alu* family is dominated by one or a few “master” elements of that family [1]. The ensuing hunt for these master element(s) led to the analysis of polymorphic *Alu* element insertions and the discovery that the frequency of these polymorphic loci varied between populations, a finding that became a cornerstone of his later independent research [4]. Mark’s later work confirmed and refined this model with the identification of secondary source elements but overall, the model remains largely consistent with observations of the evolutionary amplification of non-LTR retrotransposons.

At the turn of the millennium, the sequencing of the human genome (and then later on of an increasing number of primate genomes) initiated a new era in Mark’s research, as it enabled him to dissect and compare the repertoire of transposable elements in species at varying levels of divergence, highlighting fluctuation in *Alu* retrotransposition dynamics between humans and chimpanzees, and the existence of stealth driver *Alu* copies that may maintain low retrotranspositional activity over extended periods of time [5]. In 2007, Mark led the study of transposable elements in the rhesus macaque genome, which was published alongside the whole-genome analysis in *Science* [6]. This was an especially important achievement highlighting his fundamental contribution in promoting the recognition of transposable elements as major components of genomes.

Mark was also very interested in the consequences of the large number of transposable element copies on genome evolution [7]. As early as 1995, he described *Alu* elements as potential sources for the evolution of microsatellites in primates. Again, the genomic era enabled Mark to perform large-scale studies of genomic variation induced by retrotranspositional activity. For example, he demonstrated the evolutionary relevance of genomic deletions generated upon insertion, a phenomenon that had previously been observed only in cell culture studies of retrotransposition. Other important contributions include the quantification of human genomic deletions mediated by recombination between pairs of *Alu* and L1 elements [8], which have caused variation in gene structure, and in some cases, are associated with genetic disorders, and the demonstration that retrotransposon-mediated sequence transduction (i.e., the process by which a retrotransposon carries flanking sequence during its mobilization) is a novel gene duplication mechanism that can lead to the evolution of gene families [9].

No description of Mark’s scientific legacy is complete without mentioning his work on use of transposable elements as phylogenetic tools. While young, polymorphic *Alu* loci can be used as tools to illuminate human population history, Mark also showed that old, fixed *Alu* loci which occurred at different time points during primate evolution represent extremely robust markers for phylogenetic applications [10]. This is exemplified by the elucidation of hominid phylogeny, which provided strong evidence supporting a sister relationship between humans and chimpanzees. Likewise, the use of diagnostic *Alu* markers allowed Mark to reconstruct highly robust phylogenies for the major primate lineages, including New World and Old World monkeys. Later on, with the exponential availability of primate genome sequences, Mark resolved the phylogenies of many groups such as guenons, gibbons, baboons and owl monkeys, to name just a few.

Overall, Mark leaves behind him an unusually broad legacy coming from a lifetime of devotion to mobile DNA research. The achievements summarized here reflect > 300 peer-reviewed publications, five patents for the use of mobile elements in forensic applications, and an h-index of 86, but still constitute merely the tip of an iceberg. His vision and ability to bridge transposable elements with genome evolution and primate evolution has been a crucial factor in changing the perception of these elements from their early vilification as “junk DNA” to their current and proven recognition as major players in genome biology and function. But beyond his scientific legacy alone, Mark, a truly social person, always viewed his fellow mobile DNA scientists as his real extended family, and in this way, he leaves an irreplaceable human imprint behind through his friends, collaborators, and trainees that will long outlast his untimely passing. In parting, we leave you with his trademark signoff instructions to “Think Positive!”.

**Figure:**
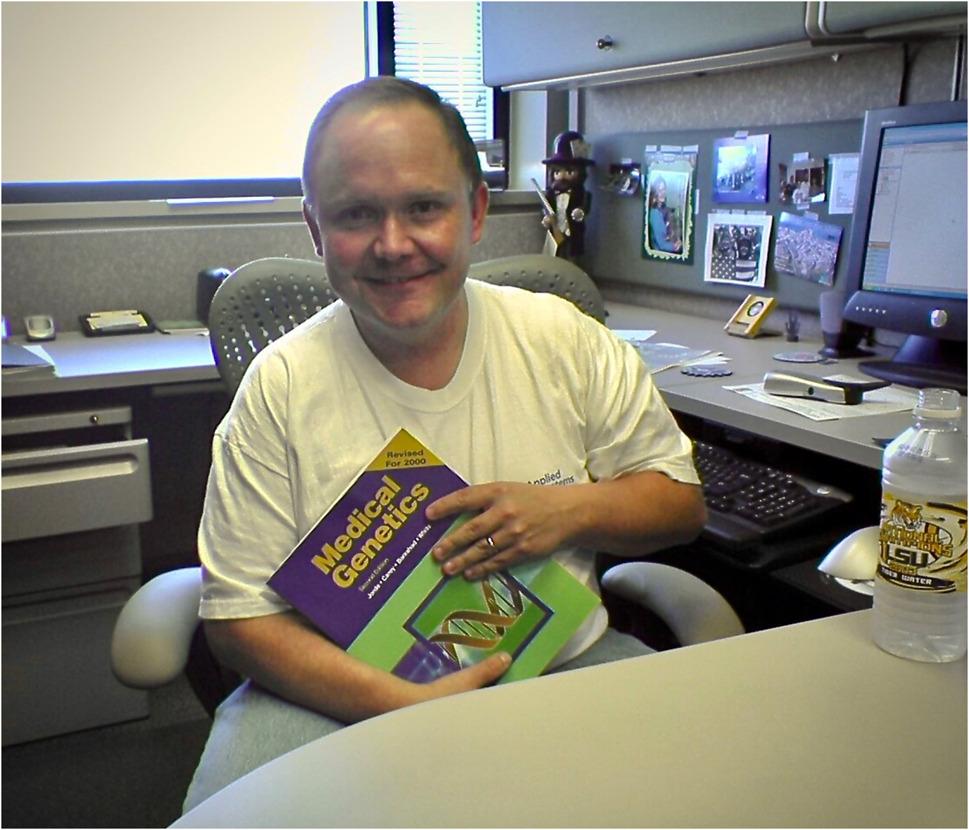
Mark in his LSU-Baton Rouge office, clearly excited about his latest publication
